# Supplementary material for: Exogenous salicylic acid, abscisic acid, and shikimic acid enhance drought tolerance in tea by modulating antioxidant defense and osmotic regulation
Source: PLoS One. 2025 Oct 16;20(10):e0331456. doi: 10.1371/journal.pone.0331456 (PMC12530598; doi:10.1371/journal.pone.0331456)
Supplement: Attachment — Submitted filename: PONE-D-25-05165.pdf [file pone.0331456.s002.pdf]

PLOS ONE

Exogenous Salicylic Acid, Abscisic Acid, and Shikimic Acid Enhance Drought Tolerance in Tea by Modulating Antioxidant Defense and Osmotic Regulation

--Manuscript Draft--

**Manuscript Number:**	PONE-D-25-05165
**Article Type:**	Research Article
**Full Title:**	Exogenous Salicylic Acid, Abscisic Acid, and Shikimic Acid Enhance Drought Tolerance in Tea by Modulating Antioxidant Defense and Osmotic Regulation
**Short Title:**	Effect of Salicylic Acid, Abscisic Acid, and Shikimic Acid on Tea under Drought stress
**Corresponding Author:**	Ajit Ghosh<br>Shahjalal University of Science and Technology<br>Sylhet, Sylhet BANGLADESH
**Keywords:**	Tea; Salicylic acid; Abscisic acid; Shikimic acid; Antioxidant enzymes; drought tolerance
**Abstract:**	Tea (Camellia sinensis), a widely cultivated crop across more than 50 countries, is highly vulnerable to drought, an abiotic stressor that severely affects its quality and yield. This study examines the potential of exogenously applied salicylic acid (SA), abscisic acid (ABA), and shikimic acid (ShA) in enhancing drought tolerance in tea plants. Tea plants underwent a four-day pre-treatment with SA, ABA, and ShA before being subjected to a 10-day drought period. Leaf samples collected on day 15 were analyzed for various physiological and biochemical markers, including antioxidant enzyme activities (POD and GST), reactive oxygen species (H2O2) levels, and contents of total chlorophyll, carotenoids, and proline. Results showed that all treatments mitigated drought-induced physiological damage, reduced oxidative stress, and elevated antioxidant enzyme activity compared to untreated control plants under drought conditions. Treated plants also exhibited higher proline and chlorophyll levels, suggesting improved osmotic regulation and photosynthetic efficiency. Among the treatments, SA demonstrated the most pronounced enhancement of drought tolerance. To clarify the functional roles and relationships of 76 genes found through literature review associated with the salicylic acid (SA) and shikimic acid (ShA) pathways under drought stress were examined using Gene Ontology (GO) enrichment, subcellular localization, and KEGG pathway analysis. These findings indicate that exogenous application of SA, ABA, and ShA can boost drought resilience in tea by strengthening antioxidant defenses and maintaining cellular integrity, presenting a promising approach to sustaining tea production in drought-prone areas.
**Order of Authors:**	Shelina Akter Sheuli, MSc
	Mir Sultanul Arafin, MSc
	Sadia Sultana, BSc
	Md. Afser Rabbi, MSc
	Ajit Ghosh, PhD
**Additional Information:**	
**Question**	**Response**
**Financial Disclosure**<br><br>Enter a financial disclosure statement that describes the sources of funding for the work included in this submission. Review the submission guidelines for detailed requirements. View published research articles from *PLOS ONE* for specific examples.	The author(s) received no specific funding for this work.

Powered by Editorial Manager® and ProduXion Manager® from Aries Systems Corporation

This statement is required for submission and **will appear in the published article** if the submission is accepted. Please make sure it is accurate.<br><br>**Funded studies**<br>Enter a statement with the following details:<br>• Initials of the authors who received each award<br>• Grant numbers awarded to each author<br>• The full name of each funder<br>• URL of each funder website<br>• Did the sponsors or funders play any role in the study design, data collection and analysis, decision to publish, or preparation of the manuscript?<br><br>Did you receive funding for this work?	
**Competing Interests**<br><br>Use the instructions below to enter a competing interest statement for this submission. On behalf of all authors, disclose any competing interests that could be perceived to bias this work—acknowledging all financial support and any other relevant financial or non-financial competing interests.<br><br>This statement is required for submission and **will appear in the published article** if the submission is accepted. Please make sure it is accurate and that any funding sources listed in your Funding Information later in the submission form are also declared in your Financial Disclosure statement.<br><br>View published research articles from *PLOS ONE* for specific examples.	he authors have declared that no competing interests exist.

**NO authors have competing interests**<br><br>Enter: *The authors have declared that no competing interests exist.*<br><br>**Authors with competing interests**<br><br>Enter competing interest details beginning with this statement:<br><br>*I have read the journal's policy and the authors of this manuscript have the following competing interests: [insert competing interests here]*<br><br>* typeset	
**Ethics Statement**<br><br>Enter an ethics statement for this submission. This statement is required if the study involved:<br><br>• Human participants<br>• Human specimens or tissue<br>• Vertebrate animals or cephalopods<br>• Vertebrate embryos or tissues<br>• Field research<br><br>Write "N/A" if the submission does not require an ethics statement.<br><br>General guidance is provided below. Consult the submission guidelines for detailed instructions. **Make sure that all information entered here is included in the Methods section of the manuscript.**	N/A

Powered by Editorial Manager® and ProduXion Manager® from Aries Systems Corporation

**Format for specific study types**<br><br>**Human Subject Research (involving human participants and/or tissue)**<br>• Give the name of the institutional review board or ethics committee that approved the study<br>• Include the approval number and/or a statement indicating approval of this research<br>• Indicate the form of consent obtained (written/oral) or the reason that consent was not obtained (e.g. the data were analyzed anonymously)<br><br>**Animal Research (involving vertebrate animals, embryos or tissues)**<br>• Provide the name of the Institutional Animal Care and Use Committee (IACUC) or other relevant ethics board that reviewed the study protocol, and indicate whether they approved this research or granted a formal waiver of ethical approval<br>• Include an approval number if one was obtained<br>• If the study involved *non-human primates*, add *additional details* about animal welfare and steps taken to ameliorate suffering<br>• If anesthesia, euthanasia, or any kind of animal sacrifice is part of the study, include briefly which substances and/or methods were applied<br><br>**Field Research**<br><br>Include the following details if this study involves the collection of plant, animal, or other materials from a natural setting:<br>• Field permit number<br>• Name of the institution or relevant body that granted permission	
**Data Availability**<br><br>Authors are required to make all data underlying the findings described fully available, without restriction, and from the time of publication. PLOS allows rare exceptions to address legal and ethical concerns. See the PLOS Data Policy and FAQ for detailed information.	Yes - all data are fully available without restriction

A Data Availability Statement describing where the data can be found is required at submission. Your answers to this question constitute the Data Availability Statement and **will be published in the article**, if accepted.<br><br>**Important:** Stating 'data available on request from the author' is not sufficient. If your data are only available upon request, select 'No' for the first question and explain your exceptional situation in the text box.<br><br>Do the authors confirm that all data underlying the findings described in their manuscript are fully available without restriction?	
**Describe where the data may be found in full sentences. If you are copying our sample text, replace any instances of XXX with the appropriate details.**<br><br>• If the data are **held or will be held in a public repository**, include URLs, accession numbers or DOIs. If this information will only be available after acceptance, indicate this by ticking the box below. For example: *All XXX files are available from the XXX database (accession number(s) XXX, XXX.).*<br>• If the data are all contained **within the manuscript and/or Supporting Information files**, enter the following: *All relevant data are within the manuscript and its Supporting Information files.*<br>• If neither of these applies but you are able to provide **details of access elsewhere**, with or without limitations, please do so. For example:<br><br>*Data cannot be shared publicly because of [XXX]. Data are available from the XXX Institutional Data Access / Ethics Committee (contact via XXX) for researchers who meet the criteria for access to confidential data.*<br><br>*The data underlying the results presented in the study are available from (include the name of the third party*	All relevant data are within the manuscript and its Supporting Information files.

and contact information or URL).<br>• This text is appropriate if the data are owned by a third party and authors do not have permission to share the data.<br><br>* typeset	
Additional data availability information:	

Manuscript Click here to access/download;Manuscript;Tea_Manuscript.docx

Exogenous Salicylic Acid, Abscisic Acid, and Shikimic Acid Enhance Drought Tolerance in Tea by Modulating Antioxidant Defense and Osmotic Regulation

Shelina Akter Sheuli[1], Mir Sultanul Arafin[1], Sadia Sultana[1], Md. Afser Rabbi[1], and Ajit Ghosh[1,*]

[1]Department of Biochemistry and Molecular Biology, Shahjalal University of Science and Technology, Sylhet 3114, Bangladesh.

[*]Corresponding author: aghosh-bmb@sust.edu

Abstract

Tea (*Camellia sinensis*), a widely cultivated crop across more than 50 countries, is highly vulnerable to drought, an abiotic stressor that severely affects its quality and yield. This study examines the potential of exogenously applied salicylic acid (SA), abscisic acid (ABA), and shikimic acid (ShA) in enhancing drought tolerance in tea plants. Tea plants underwent a four-day pre-treatment with SA, ABA, and ShA before being subjected to a 10-day drought period. Leaf samples collected on day 15 were analyzed for various physiological and biochemical markers, including antioxidant enzyme activities (POD and GST), reactive oxygen species (H_2O_2) levels, and contents of total chlorophyll, carotenoids, and proline. Results showed that all treatments mitigated drought-induced physiological damage, reduced oxidative stress, and elevated antioxidant enzyme activity compared to untreated control plants under drought conditions. Treated plants also exhibited higher proline and chlorophyll levels, suggesting improved osmotic regulation and photosynthetic efficiency. Among the treatments, SA demonstrated the most pronounced enhancement of drought tolerance. To clarify the functional roles and relationships of 76 genes found through literature review associated with the salicylic acid (SA) and shikimic acid (ShA) pathways under drought stress were examined using Gene Ontology (GO) enrichment, subcellular localization, and KEGG pathway analysis. These findings indicate that exogenous application of SA, ABA, and ShA can boost drought resilience in tea by strengthening antioxidant defenses and maintaining cellular integrity, presenting a promising approach to sustaining tea production in drought-prone areas.

Keywords

Tea, Salicylic acid, Abscisic acid, Shikimic acid, Antioxidant enzymes, drought tolerance.

Introduction

Tea (*Camellia sinensis*) is a perennial evergreen shrub native to East Asia, primarily grown for its leaves, which are processed into various types of tea, including green, black, and oolong. This plant thrives in tropical and subtropical climates, requiring specific conditions such as well-drained soil, adequate rainfall, and moderate temperatures. Tea cultivation plays a significant role in the economies of many countries, particularly in regions like China, India, Bangladesh, Sri Lanka, and Kenya, where millions of livelihoods depend on it (Zakir, 2017;Hajiboland, 2017). However, the increasing impact of climate change, particularly global warming and rising non-agricultural water demands, poses a severe threat to tea production. Drought conditions are expected to significantly reduce tea growth, yield, and quality. Studies have shown that drought can reduce tea production by 14-33% and lead to plant mortality rates of 6-19% [illegible]a and Goswami, 2021). In Bangladesh, drought has been associated with reduced tea yields, which is critical since the country exports around 18 million kilograms of tea annually, contributing about 0.81% to its GDP [illegible]mad and Hossain, 2013). Specific estates in Bangladesh experienced yield declines between 7.72% and 12.52% over three consecutive years due to drought (Das et al 2021). Similarly, in China, drought conditions have been estimated to cause an 11% to 35% reduction in tea production, leading to fluctuations in tea prices and economic losses (Zhao *et al.*, 2022;Zhao *et al.*, 2021).

One major consequence of drought stress is oxidative damage, which disrupts the physiological and biochemical processes in tea plants, causing significant losses in quality and yield [9]. During drought, the imbalance in the production and scavenging of reactive oxygen species (ROS) in plant cells results in oxidative stress, leading to cellular damage and programmed cell death (PCD), which reduces plant productivity (Sachdev *et al.*, 2021; Hasanuzzaman *et al.*, 2020). The balance between ROS production and scavenging is essential for maintaining redox biology and regulating plant growth and development ;Hasanuzzaman *et al.*, 2019). Under drought stress, tea plants adapt by triggering physiological and biochemical responses, such as osmotic pressure regulation, enhanced antioxidant activity, and hormone regulation. Plants' defense against oxidative stress involves enzymatic antioxidants, including superoxide dismutase (SOD), catalase (CAT), ascorbate peroxidase (APX), and glutathione reductase (GR), as well as non-enzymatic antioxidants such as ascorbic acid (AsA), glutathione (GSH), and flavonoids (Yang *et al.*, 2021; Hasanuzzaman *et al.*, 2020).

SOD is the first line of defense against ROS, converting superoxide into H_2O_2, which is then broken down into water by CAT, APX, and glutathione peroxidase (GPX). Non-enzymatic antioxidants, such as ascorbate and GSH, act as redox buffers within cells. GSH, a thiol-containing antioxidant, can be oxidized by ROS to form glutathione disulfide (GSSG), with NADPH as the electron donor [14]. The antioxidant defense system is crucial for plants' adaptation to drought stress. Several studies have documented drought stress responses in various crops. For instance, *Vigna radiata* seedlings exposed to drought showed reduced AsA/DHA and GSH/GSSG ratios but higher APX, GR, GPX, and GST activities, improving their tolerance to oxidative damage [15]. [illegible]milarly, drought-stressed *Brassica napus* exhibited increased phenolic content and upregulation of CAT and POD [16]. *Sorghum bicolor* cultivars subjected to drought displayed elevated H_2O_2 levels and SOD and APX activities, with the M-81E cultivar showing higher tolerance [17]. *Zea mays* and *Solanum lycopersicum* exposed to drought showed a rise in GSH content and enhanced antioxidant activity (Hussain *et al.*, 2019;Rady *et al.*, 2020).

Drought stress triggers a coordinated network of plant hormones and metabolites that regulate physiological and biochemical adaptations essential for survival. Key hormonal signaling pathways modulate stomatal closure to reduce water loss, while influencing root development to enhance water uptake from deeper soil layers. Salicylic acid, shikimic acid, and abscisic acid are three common [illegible]tabolites in plants. Salicylic acid (SA) is a phenolic compound involved in regulating plant growth and development, as well as plant responses to both biotic and abiotic stress [illegible]an *et al.*, 2015). SA has been shown to mitigate the negative effects of drought on chlorophyll content by regulating chlorophyll biosynthesis and reducing degradation, thereby maintaining photosynthetic activity under water-deficit conditions [illegible]an *et al.*, 2015). Exogenous application of SA can enhance the water potential gradient, facilitating better mineral uptake and reducing the adverse effects of drought stress. Similarly, shikimic acid (ShA), a precursor in the biosynthesis of aromatic amino acids and secondary metabolites in plants, plays a key role in producing phenolic compounds, which act as antioxidants and signaling molecules [illegible]ancenia Santos-Sánchez *et al.*, 2019). ShA resulted in increased proline levels in plants subjected to osmotic stress. This effect was attributed to enhanced activity of proline biosynthetic enzymes, particularly P5CS (Δ1-pyrroline-5-carboxylate synthetase), which is critical for proline synthesis under stress conditions [25]. Finally, abscisic acid (ABA) is another critical plant hormone that plays a significant role in stress responses, particularly drought. Exogenous ABA application

promotes the accumulation of osmolytes such as proline and soluble sugars, which help plants maintain cellular turgor and stabilize proteins under drought conditions [26]. ABA also regulates stress-responsive genes, including those encoding late embryogenesis abundant (LEA) proteins and dehydrins, which protect cellular structures and modulate water balance [27].

Given the increasing threat of drought to agriculture, particularly tea cultivation, finding effective strategies to mitigate these impacts is crucial. Enhancing tea plants' drought tolerance is essential for ensuring their survival and maintaining productivity in water-scarce environments. The main objective of this study is to establish SA, ShA, and ABA as effective inducers for tea plants against drought stress. While SA and ABA are well-established inducers against drought, their use as inducers specifically for tea plants is less common. Additionally, there is relatively little research on ShA as an inducer. In this study, the exogenous application of SA, ABA, and ShA was analyzed to improve the antioxidant defense system of tea plants under drought conditions. The effectiveness of these compounds in reducing the adverse effects of drought stress on tea biomass and enhancing antioxidant enzyme production was analyzed. By understanding the role of these compounds, we hope to contribute to sustainable tea production in the face of growing environmental challenges.

Methods and materials

Pot Experiment

To evaluate the potential of SA, ABA, and ShA for ameliorating drought stress in tea leaves, a pot experiment was conducted in the ~~general~~ laboratory with rain exclusion shelter of Biochemistry and Molecular Biology Department, Shahjalal University of Science and Technology, Sylhet (Average temperature = 25^0 C). 4-month-old seedlings of tea plants of BTS1(BT1 × TV1) variants were obtained from the Lakka Tura Tea Garden, Sylhet. These seedlings were taken and sown in p[illegible]. The experiment was performed using a completely randomized design (CRD) with tw[illegible] replicates of each treatment. Pots with draining holes were watered with 15 ml of and kept for regaining stability to the new environment. We used 16 plastic pots to plant the seedlings one in each pot. After 3 days of potting, leaf samples were collected for various chemical assays as initial data (Day 0), and from the same day, foliar application of 15 ml of SA (4mM), ShA (4 mM), and ABA (100 µM) was done continuously for 4 days to the selected plants. This dose of SA and ABA was selected based on the previous experiment (Youssef *et al.*, 2022;Liu *et al.*, 2013). A

concentration of 4 mM shikimic acid was employed in this study, which exceeds the concentration of 160 ppm typically used in previous studies where seeds were soaked in the inducer solution. In contrast, the present experiment utilized a foliar spray method for the application [30][illegible] control group was sprayed with an equal volume of distilled water during this time. After 2 days of foliar spraying, Drought stress was applied by withholding water supply on drought treatment groups for the next 10 days. The watering of the drought-stressed group was completely stopped. The control and non-stressed group were kept well-watered with 15 ml of distilled water regularly every day at the same time ~~using a measuring cylinder and foliar spray~~ (Table 1). After 10 d of treatment, the plant leaves were collected for biochemical and enzymatic analysis (Day 15).

Table 1: Treatments in the pot experiment

Sl no	Symbol	Treatment
1	Control	Irrigated control (Non-treated with any foliar spray)
2	Drought	Drought stress control (Non-treated seedlings under drought stress)
3	ABA	Abscisic acid control (Foliar spray of ABA)
4	ABA+D	Plants sprayed with ABA under 10 days drought stress
5	SA	SA control (Foliar spray of SA)
6	SA+D	Foliar spray of SA under 10 days drought stress
7	ShA	ShA control (Shikimic acid foliar spray)
8	ShA+D	Plants sprayed with shikimic acid under 10 days drought stress

Estimation of Photosynthetic Pigments

Chlorophyll "a" and "b" content was extracted and measured (in µg/ml FW) using a UV–Vis recording spectrometer at 663 and 646 nm following the method described previously [31]. Distilled water was mixed with reagent-grade acetone in a ratio of 2:8 for 80% acetone. Fresh leaf samples were collected and homogenized using mortar and pestle with fine sand. 1.5 ml of 80% acetone was used as the extractant solvent. The homogenized sample was then transferred in Eppendorf tubes and centrifuged for 15 minutes, 4000 g at 4^{0}[illegible]emperature. After centrifugation, the supernatant was collected, which was then analyzed Chlorophyll-a, Chlorophyll-b, and carotenoid content by detecting their absorbance with a UV-visible light spectrophotometer. All samples being analyzed were read at 646 nm,663 nm, and 470 nm [31].

Measurement of Proline Content

Proline contents were quantified by using the previously described protocol [32]. 200 mg of ground fresh leaf tissue was homogenized in 3 ml of 100 mM phosphate buffer (NaH_2PO_4; $NaHPO_4$) pH 7.8. The homogenate was centrifuged, and the supernatant was stored as a total crude extract at -20°C. To estimate the proline content, 50 µl of crude mix was added to 1 ml of reaction mixture containing 250 µl of 3% sulphosalicylic acid, 250 µl of acetic acid, and 500 µl of 2.5% ninhydrin solution. The mixture was boiled in a boiling water bath for 15 min and cooled down on ice for 5 min. The absorbance value was read at 520 nm. The proline content was estimated using a standard curve prepared with varying ranges (0–1 mg/ml) of proline concentration.

Measurement of Hydrogen Peroxide Content

Levels of hydrogen peroxide from tea leaves were quantified by following the previously described protocol [33]. The leaf samples were weighed, homogenized with 1 ml of 0.1% trichloroacetic acid (TCA), and centrifuged. The leaf extract supernatant (200 µl) was mixed with 300 µl of 100 mM potassium phosphate buffer (KH_2PO_4, K_2HPO_4, pH 7.8) and 500 µl of reagent (1 M KI). The blank control consisted of 200 µl of 0.1% TCA, 300 µl of 100 mM potassium phosphate buffer (pH 7.8), and 500 µl of reagent (1 M KI). After 1 hour of reaction in darkness, the absorbance was measured at 390 nm. The amount of H_2O_2 was calculated using a standard curve prepared with known concentrations of H_2O_2.

Extracting total protein for Enzyme Activity

To conduct enzyme activity from total protein extract, proteins were isolated in their native form with appropriate care. All the steps should be carried out at 4°C. Fresh plant tissue (~200 mg) was ground and homogenized in an ice-cold extraction buffer containing 100 mM potassium phosphate buffer, pH 7.0, 50% glycerol, 16 mM $MgSO_4$, and 0.5 mM PMSF, like denaturing conditions [34]. The homogenate is centrifuged at 12,000 g for 30 minutes at 4°C. The supernatant fraction is employed as a crude extract and quantified by the Bradford method [35].

Peroxidase Activity

Peroxidase (POD, EC: 1.11.1.7) activity was assessed according to the previous method [36]. The reaction mixture contained K-P buffer (25 mM, pH 7.0), guaiacol (0.05%), H_2O_2 (10 mM), and the enzyme solution (20 µL) (final volume 1000 µL). An increase in absorbance was recorded at

470 nm for 2 min, and the extinction coefficient of 26.6 mM^{-1} cm^{-1} was used for the calculation of activity.

GST Activity

GST activity was determined spectrophotometrically. The reaction mixture contained 100 mM Tris-HCl buffer (pH 6.5), 1.5 mM GSH, 1 mM 1-Chloro-2,4-dinitrobenzene (CDNB), and 20µl protein solution in a final volume of 1 ml. The increase in absorbance was measured at 340 nm for 2 minutes. The activity was calculated using the extinction coefficient of 9.6 mM^{-1} cm^{-1} [36].

Subcellular Localization, KEGG Pathway, and Gene Enrichment Analysis

A total of 76 genes (listed in Supplementary File S1) were selected through an extensive literature review to investigate their involvement in salicylic acid (SA) and shikimic acid (ShA) signaling pathways under drought stress conditions. The gene sequences were retrieved from the NCBI database. To analyze these genes, data was generated using the Database for Annotation, Visualization, and Integrated Discovery (DAVID) *(https://davidbioinformatics.nih.gov/)* (Supplementary File S1). A circular diagram was constructed using the web-based tool Chiplot *(https://www.chiplot.online/)*. Additionally, a chord diagram was generated with OriginLab *(https://www.originlab.com/)*. Furthermore, a dot plot denoting subcellular localization and the KEEG Pathway of the selected genes was created using STRING *(https://string-db.org/)*.

Statistical Analysis

The data were subjected to a thorough statistical analysis to evaluate mean differences across various treatment groups. Initially, the variances of the two samples were assessed using the F-test. This step was crucial for ensuring that the assumptions of the subsequent statistical tests were met. Following the F-test, an independent one-tailed Student's t-test was employed to compare the mean values of the control group against those of the different treatment groups. To further ascertain whether there were significant differences in the percentage changes among the treatments, the Least Significant Difference (LSD) method was applied. This method allows for the identification of specific treatment pairs that exhibit significant differences while maintaining an overall significance threshold of $P < 0.05$, as predetermined in the study design. This multi-step

analytical approach ensures robust conclusions regarding the effects of various treatments on the measured outcomes.

Result

Exogenous Treatments Reduce Drought-Induced Morphological Damage

A marked difference in the physical condition of plants when subjected to drought stress, both with and without the application of specific chemical treatments (Fig. 1). Before the imposition of drought stress, plants showed no visible signs of physical damage, which suggests a baseline of normal physiological condition across all groups (Fig. 1, upper panel). However, the imposition of drought stress alone led to substantial physical deterioration. These changes were primarily observed in their leaf water content, color, and structural integrity, which are often indicators of overall plant health and drought resilience (Fig. 1, lower panel). In contrast, plants that received treatments with SA, ABA, or ShA either alone or in combination with drought stress, exhibited significantly less physical damage (Fig.1, lower panel). This result highlights the protective role of these treatments against drought stress. The treated plants subjected to drought stress showed minimal and largely negligible changes in leaf color and structure when compared to their untreated, drought-stressed counterparts. The minimal physical damage observed in treated plants suggests that SA, ABA, and ShA may contribute to enhanced drought tolerance by mitigating some of the adverse effects of water deficit on plant physiology.

Exogenous Treatments Safeguarded Photosynthetic Pigments

The application of external treatments played a crucial role in preserving photosynthetic pigments, such as chlorophyll and carotenoids, in tea plants exposed to drought stress. All treated plants showed an increase in total chlorophyll content indicating that the treatments were effective in supporting photosynthetic pigment retention. In contrast, no significant change in chlorophyll content was observed in the control group that remained well-irrigated and untreated, suggesting stable pigment levels in the absence of stress. Notably, the drought-stressed group that did not receive any treatment experienced a sharp decrease in chlorophyll content by 20%, underscoring the detrimental impact of drought stress on chlorophyll preservation. However, plants subjected to drought stress and treated with SA, ABA, and ShA showed a noticeable increase in chlorophyll content by 15.45%, 10.29%, and 13%, respectively from Day 0. Non-stressed but treated plants

also displayed significant chlorophyll increases, with SA, ABA, and ShA leading to rises of 38.41%, 18.32%, and 31.81%, respectively (Fig. 2A). These results highlight that the exogenous application of SA, ABA, and ShA not only helped maintain higher chlorophyll levels in drought-stressed plants but also enhanced pigment levels in control plants. These findings suggest that these treatments could be valuable tools in protecting photosynthetic efficiency and enhancing drought resilience in plants.

Same kind of pattern was also observed in case of carotenoid. Treated plants exhibited carotenoid increaseswhile the untreated, irrigated control group showed no significant change (Fig. 2B). In contrast, the untreated drought-stressed group experienced a 16.98% from day 0 decrease in carotenoid levels. Drought-stressed plants treated with SA, ABA, and ShA showed increases of 13.35%, 8.75%, and 12.06%, respectively from day 0. Additionally, non-stressed treated plants demonstrated significant carotenoid increases of 35%, 16.15%, and 24.71%. These findings indicate that SA, ABA, and ShA are effective in both protecting carotenoids under drought conditions and enhancing carotenoid levels in non-stressed plants (Fig. 2B).

Exogenous Application of Plant Hormones Mitigates Drought-Induced Damage through Enhanced Proline Levels

To evaluate the role of exogenously applied compounds in mitigating oxidative damage from drought stress, we measured proline concentrations in tea leaves. Proline, an amino acid associated with stress tolerance, was analyzed to understand how treatments with SA, ABA, and ShA influence its accumulation under drought conditions. The application notably increased proline content in drought-stressed plants, with rises of 63.46%,55.23%, and 52.81%, respectively from day 0. These increases were significantly higher than those in the untreated drought-stressed plants, which showed a 40.37% from day 0 increase in proline content. This difference suggests that these treatments substantially enhance proline accumulation, likely aiding in oxidative stress management. On the other hand, proline content also increased slightly in non-stressed plants treated with SA, ABA, and ShA, with modest gains of 17.71%, 14.38%, and 11.13%, respectively from day 0. (Fig. 2C). This indicates that while these treatments can stimulate proline production without stress, their effect is more pronounced under drought conditions. The control group, which was untreated and non-stressed, showed no significant change in proline levels, reinforcing that these increases are treatment dependent. So, SA, ABA, and ShA can effectively promote proline

accumulation under drought stress, potentially contributing to enhanced stress resilience in tea plants.

Exogenous Application of Plant Hormones Reduces ROS level (H_2O_2)

To evaluate the effectiveness of exogenously applied treatments in reducing oxidative stress in tea plants under drought conditions, we measured H_2O_2 levels as an indicator of ROS accumulation, which is typically elevated during drought stress and can lead to cellular damage. Drought-stressed plants treated with SA, ABA, and ShA exhibited significantly lower increases in H_2O_2 levels compared to untreated drought-stressed plants (Fig. 2D). Treated plants showed an increase of 21.36%, 23.20%, and 27.95% from day 0 in H_2O_2 levels, respectively. In contrast, the untreated drought-stressed group experienced a marked increase of nearly 49.04% in H_2O_2 levels from day 0, highlighting the heightened oxidative stress faced by these plants. This comparison underscores the protective effect of the exogenous treatments, as the reductions in H_2O_2 levels in the treated groups suggest better management of oxidative stress, while the treated non-stressed plants showed minor increases in H_2O_2 levels of 10.87%, 8.37%, and 7.73% with SA, ABA, and ShA, respectively from day 0, this indicates the signaling role of these inducers, as the slight increase in H_2O_2 is associated with defense-related signal transduction.

Exogenous Application of Plant Hormones Enhances Antioxidant Defense Mechanisms

No significant changes in the average activity of POD in control groups over time, with levels remaining stable at 1.30%. However, the application of SA, ABA, and ShA resulted in a marked reduction in POD enzyme activities in well-watered tea plants. This suggests that these treatments may downregulate the antioxidant defense mechanisms under non-stress conditions, possibly as a means of resource allocation. Conversely, in drought-stressed plants, the subsequent application of these treatments led to significant increases in POD activity, with enhancements of 143.46%, 44.64%, and 50.27% for SA, ABA, and ShA, respectively. This indicates that the treatments effectively activated the antioxidant defense system in response to drought stress, helping to mitigate oxidative damage. In comparison, untreated drought-stressed plants showed only a modest increase of 21.63% in POD activity, highlighting the limited capacity of their antioxidant response in the absence of treatment. These percentages were also derived from the average values recorded from Day 0 to Day 15 (Fig. 3A). These findings underscore the dual role of exogenous

treatments in modulating POD activity, enhancing the enzyme's defensive role against oxidative stress in drought conditions while potentially suppressing its activity when plants are well-watered. Similarly, the average GST activity in the control groups remained relatively stable, with a slight decrease of 1.43% calculated from the average values over this period. Application of SA, ABA, and ShA led to modest increases in GST activity in well-watered tea plants, with increases of 19.73%, 12.92%, and 13.38%, respectively. These percentages were also derived from the average values recorded from Day 0 to Day 15, suggesting that these treatments may enhance the detoxification pathway, albeit to a minor extent, under non-stress conditions. Conversely, when applied to drought-stressed plants, these treatments resulted in significant increases in GST activity, with enhancements of 159.82%, 139.81%, and 120.78% for SA, ABA, and ShA, respectively, indicating that the treatments effectively activated the detoxification processes in response to oxidative stress induced by drought. In comparison, untreated drought-stressed plants experienced only a modest increase of 68.74% in GST activity, highlighting their limited detoxification capacity without treatment (Fig. 3B). These results underscore the essential role of exogenous treatments in modulating GST activity. While they promote only minor enhancements in well-watered conditions, they significantly boost the enzyme's ability to combat oxidative stress in drought-stressed tea plants.

Salicylic Acid Outperforms Abscisic Acid and Shikimic Acid as an Inducer

To compare the effects of the inducers, a comparison was made based on the extent to which each inducer altered the biochemical parameters relative to the untreated control group at day 15 (Fig. 4A). In non-drought conditions, inducer treatments increased chlorophyll and carotenoid levels. SA demonstrated the most significant enhancement in chlorophyll and carotenoid content compared to other treatments. Chlorophyll levels increased by 37.61%, and carotenoid levels rose by 34.04% with SA treatment, surpassing the improvements observed with ABA and ShA. In contrast, treatments involving H_2O_2, proline, POD, and GST showed minimal changes compared to the control. Among these SA again demonstrated the most substantial effect, with increases of 10.2%, 18.05%, 11.15%, and 20.87% in H_2O_2, proline, POD, and GST levels, respectively.

To compare the effects of inducers under drought stress versus drought stress alone, a comparison was made based on the extent to which the inducers influenced various biochemical parameters, relative to the untreated control group, at day 15 (Fig. 4B). Inducer treatments significantly

improved chlorophyll and carotenoid levels, with SAD showing the most substantial increases (14.65% and 12.39%, respectively), followed by ShAD and ABAD. In contrast, drought-stressed plants without inducers exhibited reductions in these parameters (chlorophyll -20.41%, carotenoids -17.95%). While drought stress elevated H_2O_2 accumulation, the inducers mitigated this effect, with SAD showing the least increase (20.78%), but drought stress without inducer exhibited the highest accumulation which was 48.48% higher than the control group. Antioxidant enzyme activities, including POD and GST, were significantly enhanced, particularly with SAD (POD 141.99%, GST 161.26%). Drought-stressed plants without inducers showed lower increases in POD and GST (64.35% and 70.19%, respectively). Proline accumulation was also higher in all stressed plants, with the most significant increase in SAD-treated plants (63.46%).

Gene Localization and Enrichment Analysis:

To investigate gene functions and associations, Gene Ontology (GO) biological and molecular process analysis, subcellular localization, and KEGG functional enrichment analysis were performed. A total of 76 genes involved in salicylic acid (SA) and shikimic acid (ShA) pathways under drought conditions were identified (Supplementary File S1). The most significant GO terms for biological and molecular processes were analyzed to provide a comprehensive overview of gene functions (Figure 5A). The relationship between GO terms and genes is illustrated in (Figures 5B and 5C) respectively. Detailed annotation of the GO enrichment results is provided in (Supplementary File S1).

GO analysis of biological processes revealed that differentially expressed genes (DEGs) were significantly enriched in categories such as activation of response to stimulus, response to chemicals, response to stress, and pigment metabolic process (Figure 5B). For molecular processes, significant enrichment was observed in proline biosynthetic processes, antioxidant activity, inorganic transmembrane transporter activity, regulation of signal transduction, and so on (Figure 5C). Subcellular localization analysis indicated that the DEGs were primarily enriched in plastids, chloroplast, cytoplasm, and other cellular compartments (Figure 6B). Furthermore, KEGG pathway enrichment analysis showed that DEGs were predominately involved in pathways mentioning phenylalanine, tyrosine, and tryptophan biosynthesis, glutathione biosynthesis, flavonoid biosynthesis, and carotenoid biosynthesis pathways (Figure 5A).

Discussion

The current study aimed to evaluate the efficacy of SA, ABA, and ShA in mitigating drought stress in tea plants. Foliar application of these compounds significantly enhanced drought tolerance, as demonstrated by improved biochemical parameters and reduced oxidative damage. Drought stress, recognized as a major limiting factor in agricultural productivity, causes severe physiological disruptions, particularly in photosynthesis, chlorophyll content, and growth parameters (Ahluwalia et al, 2021). The foliar application of plant growth regulators, such as SA, ABA, and ShA, has been previously studied for their roles in modulating plant responses to abiotic stress (Sabagh *et al.*, 2021). They are involved in critical metabolic pathways, including the glyoxylate cycle and gluconeogenesis, which support energy metabolism under stress conditions. Their capacity of to regulate plant metabolic processes further reinforces their role as a key modulator in stress tolerance (Miura and Tada, 2014;Jiang and Zhang, 2001).

The reduction in chlorophyll content, often observed under drought conditions due to pigment degradation and photosynthetic enzyme denaturation, was alleviated by the treatments. Inducers-treated plants maintained higher levels of chlorophyll a and b, indicating that inducers not only mitigate chlorophyll degradation but also maintain photosynthetic efficiency under water-limited conditions (Fig. 2A) aligned with previous studies showed that exogenous application of SA and ABA mitigate drought-induced damage by regulating chlorophyll biosynthesis and inhibiting chlorophyll degradation (Khan *et al.*, 2015; Jiang and Zhang, 2001;Nazar *et al.*, 2015). A similar outcome was also observed with shikimic acid (ShA) in a previous study involving tomatoes [30]. Proline, a well-established osmoprotectant, was significantly accumulated in SA, ABA, and ShA-treated plants under drought conditions (Fig. 2C). This increase in proline content is crucial for maintaining cellular turgor, osmotic balance, and protecting cellular structures during water-deficit stress aligning with previous studies where it was proven exogenous SA and ABA applications significantly enhance proline accumulation, thereby improving drought tolerance (Urmi *et al.*, 2023 ;Kaur and Asthir, 2020). Similar trends were demonstrated for the ShA treatment in proline metabolism (Yan *et al.*, 2023;Liang *et al.*, 2013).

In addition to the proline response, the reduction in H_2O_2 levels in treated plants underscores the role of SA, ABA, and ShA in mitigating oxidative stress (Fig. 2D). H_2O_2, a reactive oxygen species

(ROS) commonly produced under drought stress, can cause significant cellular damage, including lipid peroxidation and protein oxidation. Untreated drought-stressed plants accumulated significantly higher levels of H_2O_2, indicative of oxidative damage, whereas plants treated with SA, ABA, and ShA exhibited reduced oxidative stress. This reduction in ROS is consistent with earlier studies (Sah et al, 2016;Hasanuzzaman *et al.*, 2020), where the involvement of SA and ABA was reported in modulating antioxidant enzyme activities, particularly superoxide dismutase (SOD), peroxidase (POD), and catalase (CAT). Our study showed that SA, ShA, and ABA-treated plants exhibited higher POD and GST activities (Fig. 3, A and B). The induction of these enzymes is critical in detoxifying ROS and protecting cellular structures from oxidative damage.

The analysis of DEGs linked to salicylic acid (SA) and shikimic acid (ShA) pathways under drought stress emphasizes their role in stress tolerance [46,47]. The overlap between the 'stress-related response' and 'response to chemical' Gene Ontology (GO) terms indicates that the activation of SA and ShA resembles the defense responses triggered by drought (Figure 5B). This interaction enhances defense mechanisms, including proline accumulation, peroxidase (POD) activity, and glutathione S-transferase (GST) activity. Key GO terms like pigment synthesis, GST activity, and proline biosynthesis share genes with 'response to stimulus' indicating their activation under combined stress and induced conditions (Figure 5C). Subcellular localization in plastids, chloroplasts, and cytoplasm supports photosynthetic roles in photosynthesis and metabolic adjustments (Figure 6B). KEGG pathway analysis further highlights involvement in antioxidant and stress signaling pathways (Figure 6A), aligning with wet lab findings and underscoring the potential of inducers to enhance drought resilience.

The reduction in morphological damage observed in the induced plants can be attributed to the enhanced activity of antioxidant enzymes and osmoprotectants, which mitigate oxidative stress. Additionally, the increase in photopigment synthesis contributes to improved photosynthetic efficiency and cellular protection. It has been demonstrated that salicylic acid (SA) and abscisic acid (ABA) play pivotal roles in regulating stomatal closure, thereby minimizing water loss under stress conditions. This stomatal regulation, in turn, helps to conserve water and maintain cellular homeostasis, further protecting the plant from environmental damage (Lim *et al.*, 2015; Jiang and Zhang, 2002; Prodhan *et al.*, 2018).

Drought stress without any inducers decreased photosynthetic pigments while still elevating stress response markers, indicating the protective effects of the tested inducers on photosynthetic

apparatus under drought conditions. To ensure the effective action of the inducers, extended molecular mechanisms were involved in mediating their effects. SA plays a crucial role in activating NPR1, a key regulator of plant defense mechanisms [51]. In its inactive form, NPR1 exists as an oligomer, stabilized by disulfide bonds. The intracellular levels of SA increase, triggering a reduction reaction that breaks the disulfide bonds in NPR1. This reduction causes NPR1 oligomers to dissociate into active monomers. These monomeric NPR1 proteins can then translocate into the nucleus, where they interact with transcription factors. Together, they bind to the promoters of various defense-related genes, activating their expression [52]. ShA is a key intermediate in the shikimate pathway, which feeds into the phenylpropanoid pathway, which is crucial for the synthesis of SA and various polyphenols (Roychoudhury, 2024;Kumar *et al.*, 2023). These polyphenols play a significant role in plant defense mechanisms. Polyphenols can directly engage in defense-related pathways, as they help modulate oxidative stress responses [55]. When pathways are activated by external or internal inducers, H_2O_2 levels may increase slightly to stimulate further defensive pathways, enhancing the plant's resistance to stress or pathogens. This balance between signaling and scavenging ensures that the plant can effectively respond to stress without suffering adverse effects from prolonged oxidative stress (Kanner, 2020,Srinivasulu *et al.*, 2018). ABA signaling in plants primarily involves PYR/PYL/RCAR receptors, PP2C phosphatase, and SnRK2 kinase. Elevated ABA levels enable the PYR/PYL/RCAR-PP2C complex formation, inhibiting PP2C and activating SnRK2. Activated SnRK2 phosphorylates transcription factors that drive the expression of ABA-responsive genes, facilitating physiological adaptations like stomatal closure to conserve water (Sah et al, 2016). Beyond this pathway, ABA-mediated mechanisms further support drought tolerance. ABP9, a bZIP family protein, enhances photosynthetic capacity under drought stress. Additionally, histone acetylation allows dynamic ABA-regulated gene expression changes, and the MAPK signaling cascade reinforces ABA-mediated drought adaptation at transcriptional and proteomic levels (Aslam *et al.*, 2022).

The present study offers novel insights into the combined and individual roles of SA, ABA, and ShA in conferring drought tolerance through multiple mechanisms, including enhanced proline synthesis, reduced ROS accumulation, and increased antioxidant enzyme activities. While SA emerged as a particularly potent modulator of drought stress responses, both ABA and ShA also contributed significantly to enhancing plant resilience. These findings not only reaffirm the potential of SA, ABA, and ShA as effective treatments for improving drought tolerance but also

provide a mechanistic understanding of how these compounds regulate key metabolic and physiological processes under stress conditions.

Conclusion

The study conducted an in-depth comparative analysis of several plant growth regulatory elements, revealing that the application of ABA, ShA, and SA significantly enhances drought tolerance in tea plants. These regulatory elements bring about beneficial changes in morphological and physiological characteristics, particularly by enhancing the activity of ROS-scavenging enzymes. These enzymes are vital for detoxifying ROS, which are produced in excess under drought conditions and can lead to cellular damage. By boosting these enzymes, the treatments mitigate oxidative stress and reinforce the plant's overall resilience to drought. The practical implications of using ABA, ShA, and SA are noteworthy, especially for rehabilitating drought-stricken lands, particularly in arid and semi-arid regions where water scarcity consistently threatens agricultural productivity.

Ethics approval and consent to participate

All experimental research on plants, including the collection of plant materials has been used by following the relevant institutional, national, and international guidelines and legislation.

Authors' contributions

AG conceived the idea, designed the experiments, validated the results, reviewed and edited the manuscript, and supervised the project. SAS, MSA, SS, and MAR performed all experiments, conducted data curation and analysis, data visualization, and wrote the original draft.

Availability of data and materials

The authors declare that all the data and plant materials will be available without restrictions. All the plant materials and the raw data used in the manuscript will be available to Dr Ajit Ghosh (aghosh-bmb@sust.edu).

Competing interests

The authors declare that there is no competing interest.

Funding

There was no specific research fund for this study.

Abbreviations

SA, Salicylic acid; ABA, Abscisic acid; ShA, Shikimic acid; POD, Peroxidase; GST, Glutathione-S-transferase; ROS, Reactive oxygen species; AsA, Ascorbic acid; GSH, Glutathione; SOD, Superoxide dismutase, CAT, Catalase; APX, Ascorbate peroxidase; GR, Glutathione reductase; GPX, Glutathione peroxidase; GSSG, Glutathione disulfide; NADPH, Nicotinamide adenine dinucleotide phosphate; NPR1, Nonexpressor of pathogenesis-related genes 1; P5CS, Δ1-pyrroline-5-carboxylate synthetase; LEA, Late embryogenesis abundant; PYR/PYL/RCAR, Pyrabactin resistance 1/Pyrabactin resistance-like/Receptor for activated protein C kinase; PP2C, Protein phosphatase 2C; SnRK2, Sucrose non-fermenting 1-related protein kinase 2; CRD, Completely Randomized Design; TCA, Trichloroacetic Acid; H_2O_2, Hydrogen Peroxide; PMSF, Phenylmethylsulfonyl Fluoride; PMS, Phosphate-Buffered Saline; K-P, Potassium Phosphate; CDNB, 1-Chloro-2,4-Dinitrobenzene; ZIP, Basic Leucine Zipper (a type of transcription factor); MAPK, Mitogen-Activated Protein Kinase; ABP9, ABA-responsive bZIP 9.

Acknowledgment

The authors extend their gratitude to the Department of Biochemistry and Molecular Biology at Shahjalal University of Science and Technology, Sylhet, Bangladesh, for providing lab facilities and logistic support.

References

1. Zakir M. Review on Tea (Camellia sinensis) Research Achievements, Challenges and Future Prospective Including Ethiopian Status. Int J For Hortic. 2017;3: 27–39.

2. Hajiboland R. Sciendo. Folia Hortic. 2017;29: 199–220. doi:10.1515/fhort-2017-0019

3. Deka N, Goswami K. Economic sustainability of organic cultivation of Assam tea produced by small-scale growers. Sustain Prod Consum. 2021;26: 111–125. doi:10.1016/j.spc.2020.09.020

4. Cheruiyot EK, Mumera LM, Ng'etich WK, Hassanali A, Wachira FN. HIGH FERTILIZER RATES INCREASE SUSCEPTIBILITY OF TEA TO WATER STRESS. J Plant Nutr. 2009;33: 115–129. doi:10.1080/01904160903392659

5. Ahmad I, Hossain M. Present Status of Bangladesh Tea Sector in Respect of World Tea: An Overview. 2013;9: 116–128.

6. Das AC, Noguchi R, Ahamed T. An Assessment of Drought Stress in Tea Estates Using Optical and Thermal Remote Sensing. Remote Sens. 2021;13: 2730. doi:10.3390/rs13142730

7. Zhao Y, Xu Y, Zhang L, Zhao M, Wang C. Adapting Tea Production to Climate Change under Rapid Economic Development in China from 1987 to 2017. Agronomy. 2022;12: 3192. doi:10.3390/agronomy12123192

8. Zhao Y, Zhao M, Zhang L, Wang C, Xu Y. Predicting Possible Distribution of Tea (Camellia sinensis L.) under Climate Change Scenarios Using MaxEnt Model in China. Agriculture. 2021;11: 1122. doi:10.3390/agriculture11111122

9. Chen S, Gao Y, Fan K, Shi Y, Luo D, Shen J, et al. Prediction of Drought-Induced Components and Evaluation of Drought Damage of Tea Plants Based on Hyperspectral Imaging. Front Plant Sci. 2021;12: 695102. doi:10.3389/fpls.2021.695102

10. Sachdev S, Ansari SA, Ansari MI, Fujita M, Hasanuzzaman M. Abiotic Stress and Reactive Oxygen Species: Generation, Signaling, and Defense Mechanisms. Antioxidants. 2021;10: 277. doi:10.3390/antiox10020277

11. Hasanuzzaman M, Bhuyan MHM, Zulfiqar F, Raza A, Mohsin S, Mahmud J, et al. Reactive Oxygen Species and Antioxidant Defense in Plants under Abiotic Stress: Revisiting the Crucial Role of a Universal Defense Regulator. Antioxidants. 2020;9: 681. doi:10.3390/antiox9080681

12. Hasanuzzaman M, Bhuyan MHMB, Anee TI, Parvin K, Nahar K, Mahmud JA, et al. Regulation of Ascorbate-Glutathione Pathway in Mitigating Oxidative Damage in Plants under Abiotic Stress. Antioxidants. 2019;8: 384. doi:10.3390/antiox8090384

13. Yang X, Lu M, Wang Y, Wang Y, Liu Z, Chen S. Response Mechanism of Plants to Drought Stress. Horticulturae. 2021;7: 50. doi:10.3390/horticulturae7030050

14. Rajput VD, Harish, Singh RK, Verma KK, Sharma L, Quiroz-Figueroa FR, et al. Recent Developments in Enzymatic Antioxidant Defence Mechanism in Plants with Special Reference to Abiotic Stress. Biology. 2021;10: 267. doi:10.3390/biology10040267

15. Nahar K, Hasanuzzaman M, Alam MdM, Rahman A, Mahmud J-A, Suzuki T, et al. Insights into spermine-induced combined high temperature and drought tolerance in mung bean: osmoregulation and roles of antioxidant and glyoxalase system. Protoplasma. 2017;254: 445–460. doi:10.1007/s00709-016-0965-z

16. Akram NA, Iqbal M, Muhammad A, Ashraf M, Al-Qurainy F, Shafiq S. Aminolevulinic acid and nitric oxide regulate oxidative defense and secondary metabolisms in canola (Brassica napus L.) under drought stress. Protoplasma. 2018;255: 163–174. doi:10.1007/s00709-017-1140-x

17. Guo YY, Tian SS, Liu SS, Wang WQ, Sui N. Energy dissipation and antioxidant enzyme system protect photosystem II of sweet sorghum under drought stress. Photosynthetica. 2018;56: 861–872. doi:10.1007/s11099-017-0741-0

18. Hussain HA, Men S, Hussain S, Chen Y, Ali S, Zhang S, et al. Interactive effects of drought and heat stresses on morpho-physiological attributes, yield, nutrient uptake and oxidative status in maize hybrids. Sci Rep. 2019;9: 3890. doi:10.1038/s41598-019-40362-7

19. Rady MM, Belal HEE, Gadallah FM, Semida WM. Selenium application in two methods promotes drought tolerance in Solanum lycopersicum plant by inducing the antioxidant defense system. Sci Hortic. 2020;266: 109290. doi:10.1016/j.scienta.2020.109290

20. Khan MIR, Fatma M, Per TS, Anjum NA, Khan NA. Salicylic acid-induced abiotic stress tolerance and underlying mechanisms in plants. Front Plant Sci. 2015;6. doi:10.3389/fpls.2015.00462

21. Liu J, Qiu G, Liu C, Li H, Chen X, Fu Q, et al. Salicylic Acid, a Multifaceted Hormone, Combats Abiotic Stresses in Plants. Life. 2022;12: 886. doi:10.3390/life12060886

22. Khalvandi M, Siosemardeh A, Roohi E, Keramati S. Salicylic acid alleviated the effect of drought stress on photosynthetic characteristics and leaf protein pattern in winter wheat. Heliyon. 2021;7: e05908. doi:10.1016/j.heliyon.2021.e05908

23. Francenia Santos-Sánchez N, Salas-Coronado R, Hernández-Carlos B, Villanueva-Cañongo C. Shikimic Acid Pathway in Biosynthesis of Phenolic Compounds. In: Soto-Hernández M, García-Mateos R, Palma-Tenango M, editors. Plant Physiological Aspects of Phenolic Compounds. IntechOpen; 2019. doi:10.5772/intechopen.83815

24. Wu S, Chen W, Lu S, Zhang H, Yin L. Metabolic Engineering of Shikimic Acid Biosynthesis Pathway for the Production of Shikimic Acid and Its Branched Products in Microorganisms: Advances and Prospects. Molecules. 2022;27: 4779. doi:10.3390/molecules27154779

25. Liang X, Zhang L, Natarajan SK, Becker DF. Proline Mechanisms of Stress Survival. Antioxid Redox Signal. 2013;19: 998–1011. doi:10.1089/ars.2012.5074

26. Muhammad Aslam M, Waseem M, Jakada BH, Okal EJ, Lei Z, Saqib HSA, et al. Mechanisms of Abscisic Acid-Mediated Drought Stress Responses in Plants. Int J Mol Sci. 2022;23: 1084. doi:10.3390/ijms23031084

27. Xiong L, Zhu J-K. Regulation of Abscisic Acid Biosynthesis. Plant Physiol. 2003;133: 29–36. doi:10.1104/pp.103.025395

28. Youssef SM, López-Orenes A, Ferrer MA, Calderón AA. Salicylic-Acid-Regulated Antioxidant Capacity Contributes to Growth Improvement of Okra (Abelmoschus esculentus cv. Red Balady). Agronomy. 2022;12: 168. doi:10.3390/agronomy12010168

29. Liu T, Zhang L, Yuan Z, Hu X, Lu M, Wang W, et al. Identification of proteins regulated by ABA in response to combined drought and heat stress in maize roots. Acta Physiol Plant. 2013;35: 501–513. doi:10.1007/s11738-012-1092-x

30. Al-Amri SM. Improved growth, productivity and quality of tomato (*Solanum lycopersicum* L.) plants through application of shikimic acid. Saudi J Biol Sci. 2013;20: 339–345. doi:10.1016/j.sjbs.2013.03.002

31. Singh S. Salicylic acid elicitation improves antioxidant activity of spinach leaves by increasing phenolic content and enzyme levels. Food Chem Adv. 2023;2: 100156. doi:10.1016/j.focha.2022.100156

32. Jain M, Aggarwal S, Nagar P, Tiwari R, Mustafiz A. A D-lactate dehydrogenase from rice is involved in conferring tolerance to multiple abiotic stresses by maintaining cellular homeostasis. Sci Rep. 2020;10: 12835. doi:10.1038/s41598-020-69742-0

33. Liu J, Sun X, Xu F, Zhang Y, Zhang Q, Miao R, et al. Suppression of OsMDHAR4 enhances heat tolerance by mediating H2O2-induced stomatal closure in rice plants. Rice. 2018;11: 38. doi:10.1186/s12284-018-0230-5

34. Islam S, Sajib SD, Jui ZS, Arabia S, Islam T, Ghosh A. Genome-wide identification of glutathione S-transferase gene family in pepper, its classification, and expression profiling under different anatomical and environmental conditions. Sci Rep. 2019;9: 9101. doi:10.1038/s41598-019-45320-x

35. Bradford MM. A rapid and sensitive method for the quantitation of microgram quantities of protein utilizing the principle of protein-dye binding. Anal Biochem. 1976;72: 248–254. doi:10.1016/0003-2697(76)90527-3

36. Singh S. Enhancing phytochemical levels, enzymatic and antioxidant activity of spinach leaves by chitosan treatment and an insight into the metabolic pathway using DART-MS technique. Food Chem. 2016;199: 176–184. doi:10.1016/j.foodchem.2015.11.127

37. Ahluwalia O, Singh PC, Bhatia R. A review on drought stress in plants: Implications, mitigation and the role of plant growth promoting rhizobacteria. Resour Environ Sustain. 2021;5: 100032. doi:10.1016/j.resenv.2021.100032

38. El Sabagh A, Mbarki S, Hossain A, Iqbal MA, Islam MS, Raza A, et al. Potential Role of Plant Growth Regulators in Administering Crucial Processes Against Abiotic Stresses. 2021;3. doi:10.3389/fagro.2021.648694

39. Miura K, Tada Y. Regulation of water, salinity, and cold stress responses by salicylic acid. Front Plant Sci. 2014;5. doi:10.3389/fpls.2014.00004

40. Jiang M, Zhang J. Effect of Abscisic Acid on Active Oxygen Species, Antioxidative Defence System and Oxidative Damage in Leaves of Maize Seedlings. Plant Cell Physiol. 2001;42: 1265–1273. doi:10.1093/pcp/pce162

41. Nazar R, Umar S, Khan NA, Sareer O. Salicylic acid supplementation improves photosynthesis and growth in mustard through changes in proline accumulation and ethylene formation under drought stress. South Afr J Bot. 2015;98: 84–94. doi:10.1016/j.sajb.2015.02.005

42. Urmi TA, Islam MM, Zumur KN, Abedin MA, Haque MM, Siddiqui MH, et al. Combined Effect of Salicylic Acid and Proline Mitigates Drought Stress in Rice (Oryza sativa L.)

through the Modulation of Physiological Attributes and Antioxidant Enzymes. Antioxidants. 2023;12: 1438. doi:10.3390/antiox12071438

43. Kaur G, Asthir B. Impact of exogenously applied ABA on proline metabolism conferring drought and salinity stress tolerance in wheat genotypes. Cereal Res Commun. 2020;48. doi:10.1007/s42976-020-00041-0

44. Yan J, Ninkuu V, Fu Z, Yang T, Ren J, Li G, et al. OsOLP1 contributes to drought tolerance in rice by regulating ABA biosynthesis and lignin accumulation. Front Plant Sci. 2023;14. doi:10.3389/fpls.2023.1163939

45. Sah SK, Reddy KR, Li J. Abscisic Acid and Abiotic Stress Tolerance in Crop Plants. Front Plant Sci. 2016;7. doi:10.3389/fpls.2016.00571

46. Khan MIR, Fatma M, Per TS, Anjum NA, Khan NA. Salicylic acid-induced abiotic stress tolerance and underlying mechanisms in plants. Front Plant Sci. 2015;6: 462. doi:10.3389/fpls.2015.00462

47. Qu X, Wang H, Chen M, Liao J, Yuan J, Niu G. Drought Stress–induced Physiological and Metabolic Changes in Leaves of Two Oil Tea Cultivars. 2019 [cited 30 Jan 2025]. doi:10.21273/JASHS04775-19

48. Lim CW, Baek W, Jung J, Kim J-H, Lee SC. Function of ABA in Stomatal Defense against Biotic and Drought Stresses. Int J Mol Sci. 2015;16: 15251–15270. doi:10.3390/ijms160715251

49. Jiang M, Zhang J. Water stress-induced abscisic acid accumulation triggers the increased generation of reactive oxygen species and up-regulates the activities of antioxidant enzymes in maize leaves. J Exp Bot. 2002;53: 2401–2410. doi:10.1093/jxb/erf090

50. Prodhan MY, Munemasa S, Nahar MN-E-N, Nakamura Y, Murata Y. Guard Cell Salicylic Acid Signaling Is Integrated into Abscisic Acid Signaling via the Ca2+/CPK-Dependent Pathway. Plant Physiol. 2018;178: 441. doi:10.1104/pp.18.00321

51. Chen J, Mohan R, Zhang Y, Li M, Chen H, Palmer IA, et al. NPR1 Promotes Its Own and Target Gene Expression in Plant Defense by Recruiting CDK8. Plant Physiol. 2019;181: 289–304. doi:10.1104/pp.19.00124

52. Zavaliev R, Dong X. NPR1, a key immune regulator for plant survival under biotic and abiotic stresses. Mol Cell. 2024;84: 131–141. doi:10.1016/j.molcel.2023.11.018

53. Roychoudhury A, editor. Secondary metabolites in environmental stress tolerance. First edition. Palm Bay, FL: Apple Academic Press; 2024.

54. Kumar K, Debnath P, Singh S, Kumar N. An Overview of Plant Phenolics and Their Involvement in Abiotic Stress Tolerance. Stresses. 2023;3: 570–585. doi:10.3390/stresses3030040

55. Zagoskina NV, Zubova MY, Nechaeva TL, Kazantseva VV, Goncharuk EA, Katanskaya VM, et al. Polyphenols in Plants: Structure, Biosynthesis, Abiotic Stress Regulation, and Practical Applications (Review). Int J Mol Sci. 2023;24: 13874. doi:10.3390/ijms241813874

56. Kanner J. Polyphenols by Generating H2O2, Affect Cell Redox Signaling, Inhibit PTPs and Activate Nrf2 Axis for Adaptation and Cell Surviving: In Vitro, In Vivo and Human Health. Antioxidants. 2020;9: 797. doi:10.3390/antiox9090797

57. Srinivasulu C, Ramgopal M, Ramanjaneyulu G, Anuradha CM, Suresh Kumar C. Syringic acid (SA) – A Review of Its Occurrence, Biosynthesis, Pharmacological and Industrial Importance. Biomed Pharmacother. 2018;108: 547–557. doi:10.1016/j.biopha.2018.09.069

Figure legends

Figure 1. Condition of plans before and after drought Stress. Before drought stress, all plants exhibited healthy leaves, vibrant color, and structural integrity, indicative of a baseline normal physiological state. No visible signs of stress or physical damage were observed, reflecting comparable initial conditions across both treated and untreated groups before the imposition of drought stress. Drought stress led to notable physical deterioration in untreated plants, as evidenced by reduced leaf water content, color loss, and compromised structural integrity. Plants treated with salicylic acid (SA), abscisic acid (ABA), and shikimic acid (ShA) displayed significantly less physical damage under drought stress conditions, suggesting enhanced drought resilience due to the protective effects of these treatments.

Figure 2. Effect of Treatments on non-enzymatic stress biomarkers in tea plants measured at Day 0 and Day 15. A) Total Chlorophyll Concentration, B) Total Carotenoid Concentration, C) Total Proline Concentration, D) Total Hydrogen Peroxide Concentration. Treatments include salicylic acid (SA), salicylic acid with drought (SA+D), abscisic acid (ABA), abscisic acid with drought (ABA+D), shikimic acid (ShA), shikimic acid with drought (ShA+D), and drought alone. Concentration is expressed in mg/ml. All values represent the mean of two replicates ± standard error of the mean (n=3). The significance level of the paired t-test is denoted by (*) and (**) with a p-value less than 0.05 and 0.01, respectively. Day 0 (blue); Day 15 (orange).

Figure 3. Effect of Treatments on enzymatic stress markers activity in tea plants measured at Day 0 and Day 15. A) Peroxidase Activity, B) glutathione S-transferase (GST). Treatments include salicylic acid (SA), salicylic acid with drought (SAD), abscisic acid (ABA), abscisic acid with drought (ABA D), shikimic acid (ShA), shikimic acid with drought (ShA D), and drought alone. GST activity is expressed in µmol/min/mg protein. All values represent the mean of two replicates ± standard error of the mean (n=3). The significance level of the paired t-test is denoted by (*) and (**) with a p-value less than 0.05 and 0.01, respectively. Day 0 (blue); Day 15 (orange).

Figure 4. Comparison of various inducer treatments on physiological and biochemical parameters in plants. The radar chart illustrates the comparative effects of salicylic acid (SA), abscisic acid (ABA), and shikimic acid (ShA) treatments on plants across two conditions: A)

Normal Conditions, with comparisons to a control, and B) Drought Stress Conditions, with comparisons to both drought-affected and control plants. Each parameter—chlorophyll, carotenoids, H_2O_2, proline, peroxidase (POD), and glutathione S-transferase (GST) activity—is normalized to the control, set at 100. Values exceeding 100 indicate an increase relative to the control, suggesting enhanced effects under treatment, while values below 100 signify a decrease, indicating reduced effectiveness compared to the control.

Figure 5: Distribution of DEGs in GO enrichment analysis. (A) The order of the circles is from the outside in. The first circle shows the most significantly enriched terms of biological process (BP) and molecular function (MF), with the scale outside corresponding to the number of genes. The second circle shows the number of background genes in the corresponding GO terms and the p-value. The third circle shows the number of DEGs. The inner circle shows the enrichment score of the corresponding GO terms. Genes are involved in SA and ShA signaling pathways with their respective GO terms. **(B)** Depicts GO terms of Biological Processes (BP), while **(C)** shows GO terms of Molecular Processes (MP). The direction of the lines indicates gene associations with specific GO terms, and the bar thickness represents the number of genes in each category.

Figure 6: KEGG pathway and Gene Ontology analysis. A bubble chart for **(A)** KEGG pathway and **(B)** Gene Ontology enrichment analysis term for the category of cellular components of identified 76 genes through literature review. The Y-Axis indicates the pathway and cellular component name; and the x-axis indicates the enriched factor. The bubble size indicates the number of genes, and the color bar indicates the FDR values.

Figure 1

Click here to access/download;Figure;Figure_1.tif

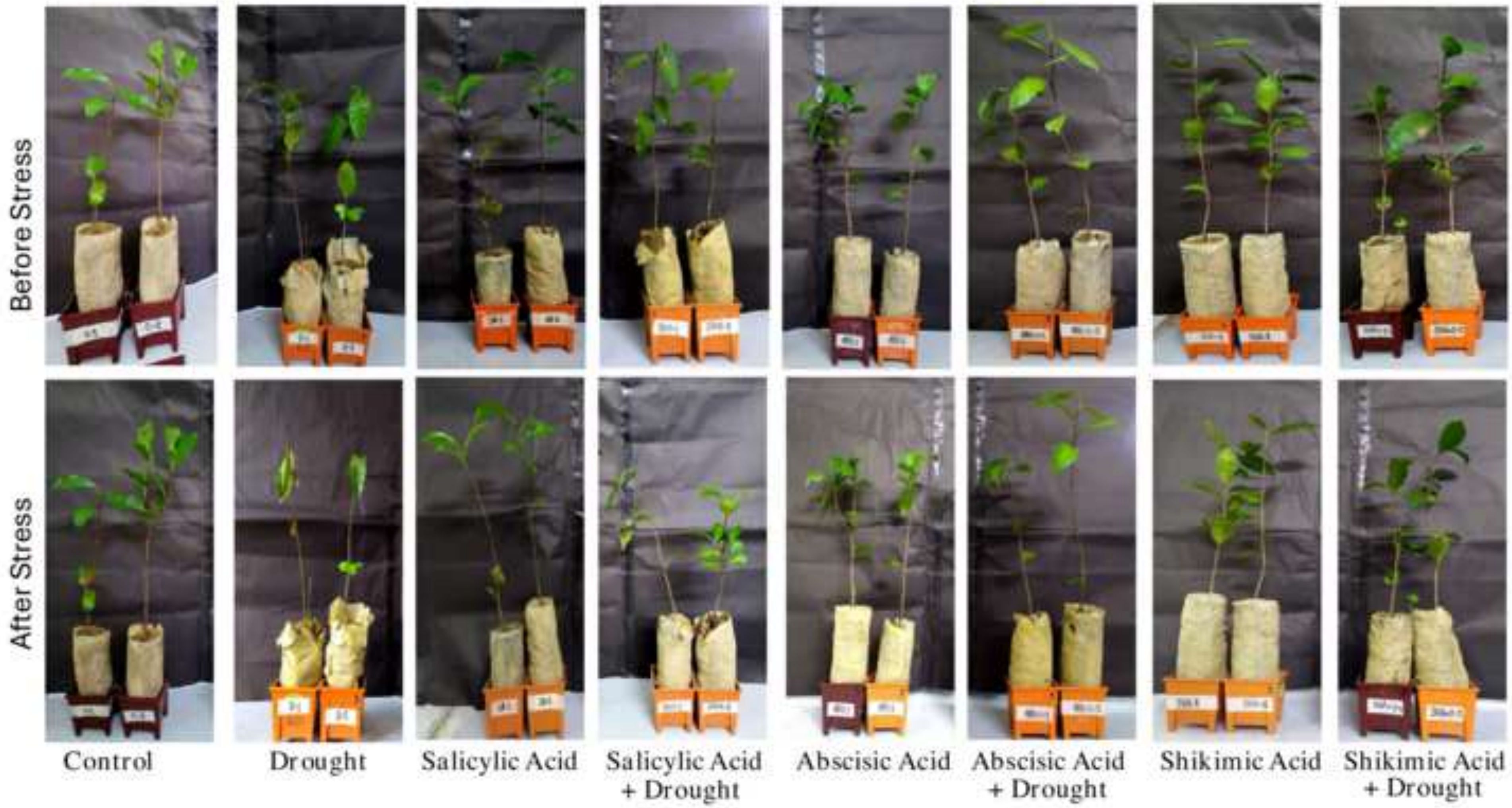

Figure 2

Click here to access/download;Figure;Figure_2.tif

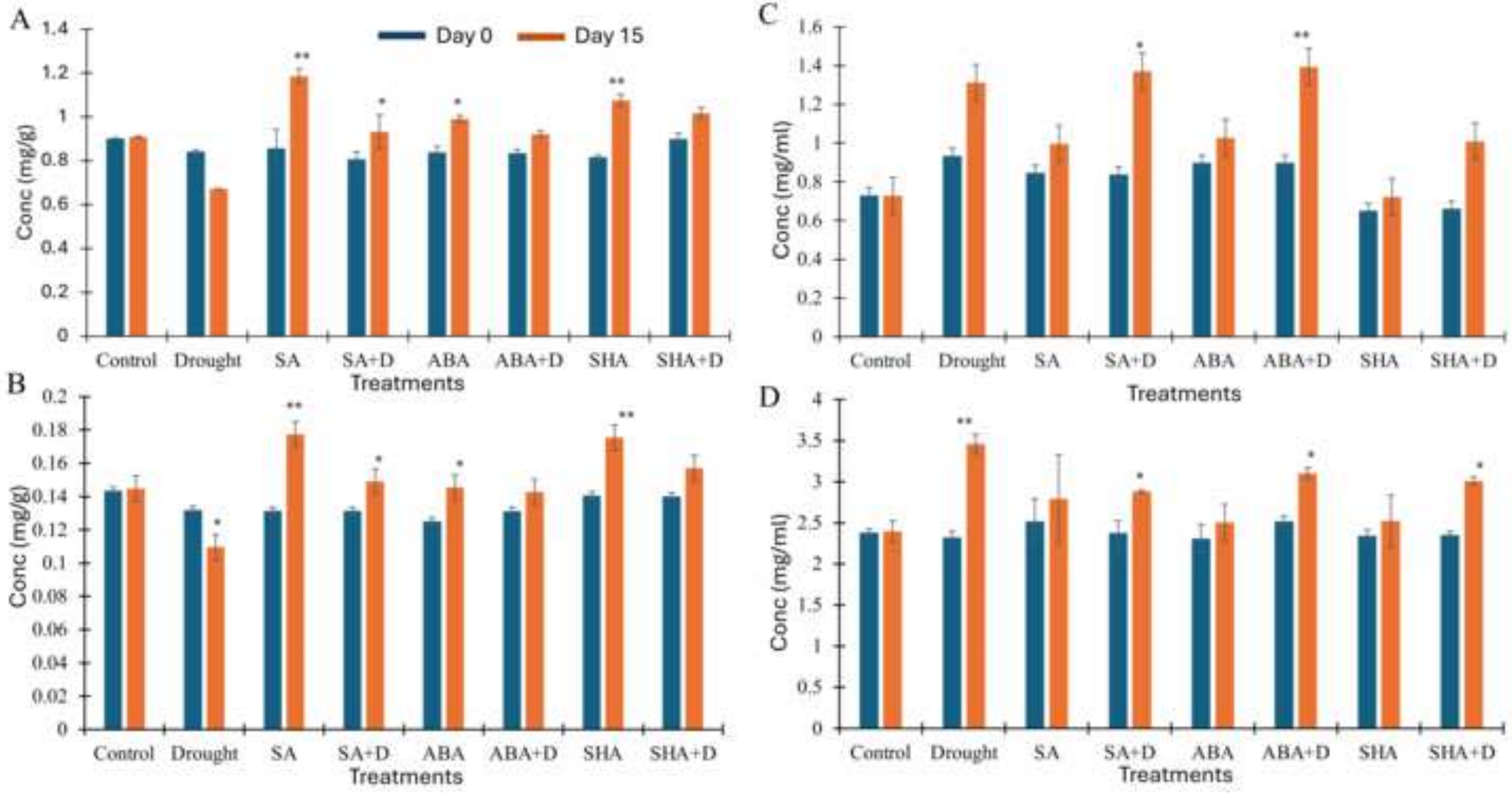

Figure 3

Click here to access/download;Figure;Figure_3.tif

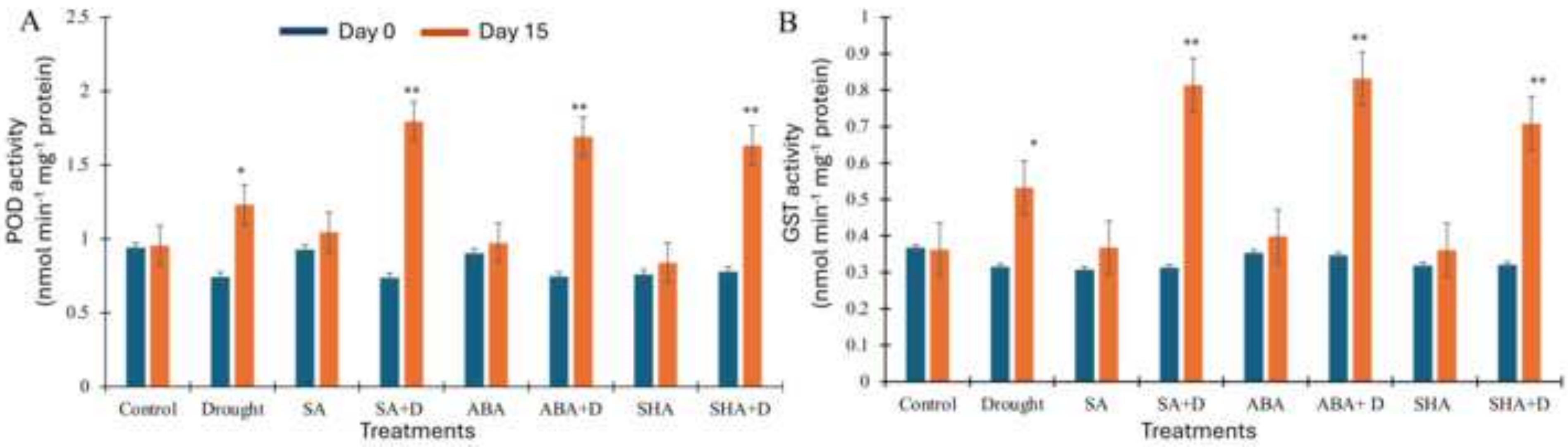

Figure 4

Click here to access/download;Figure;Figure_4.tif

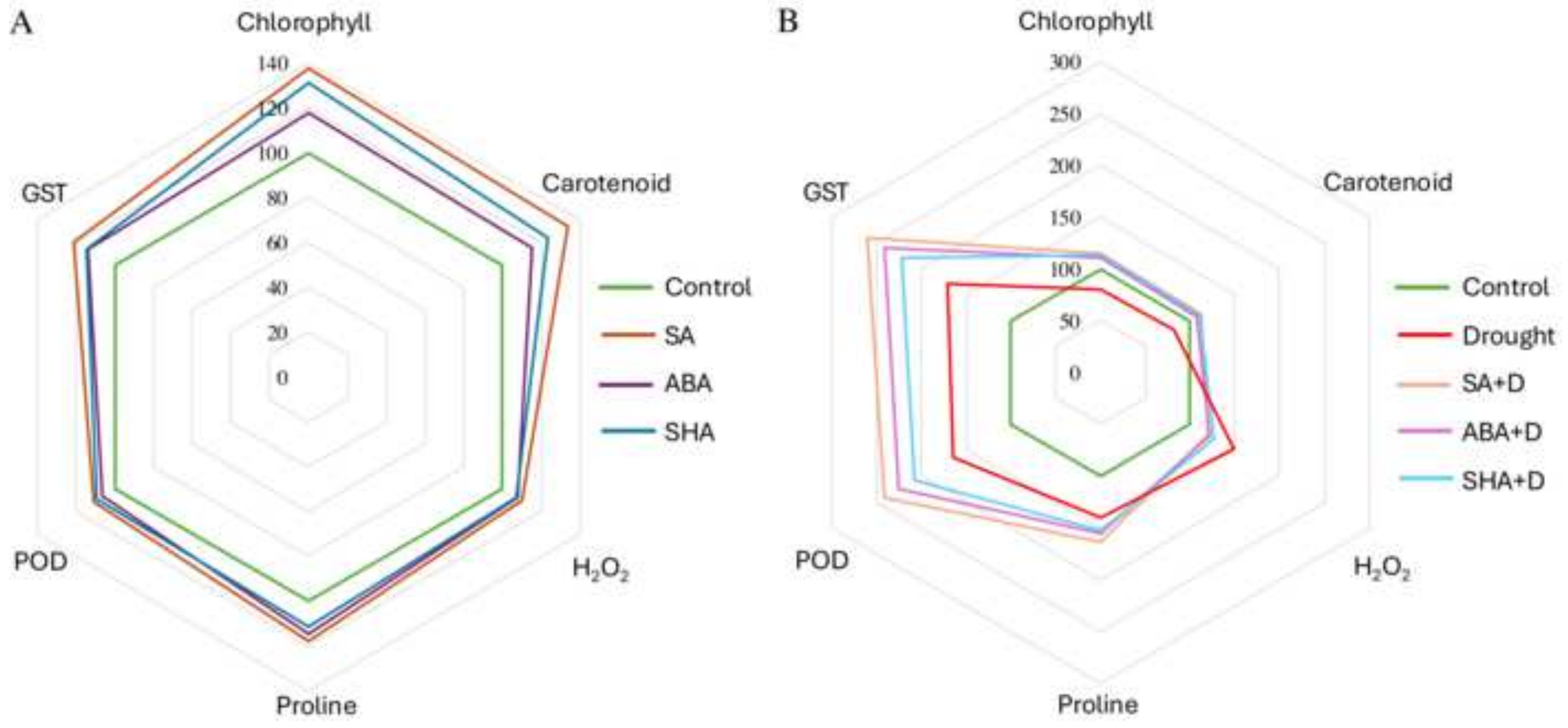

Figure 5

Click here to access/download;Figure;Figure_5.tif

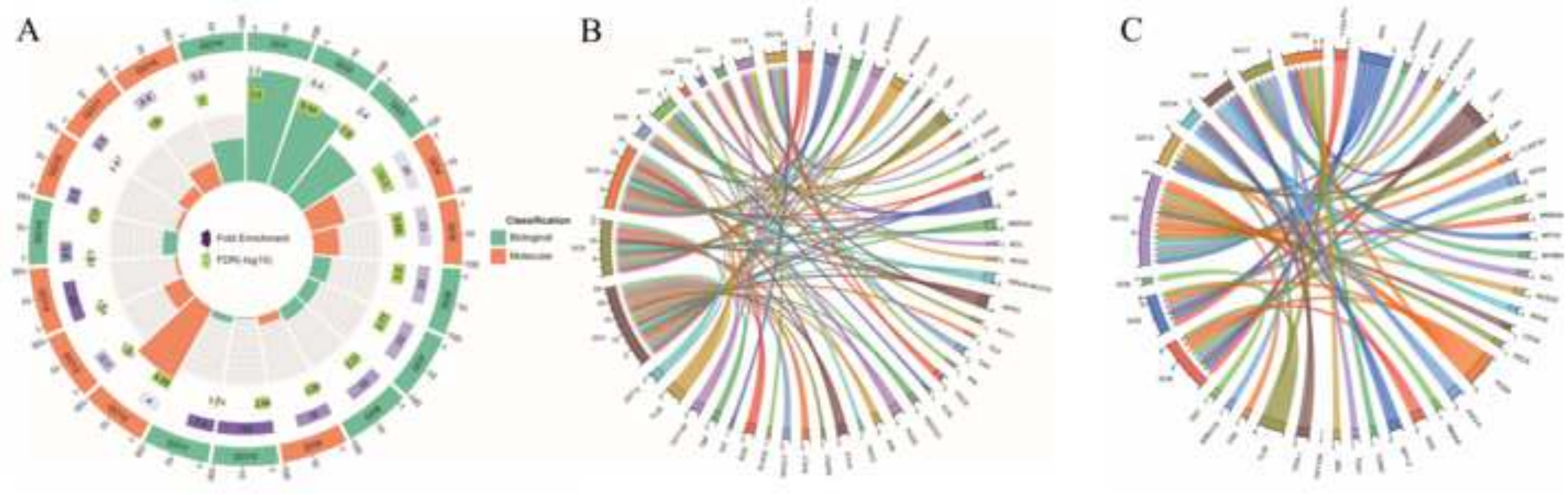

Figure 6 Click here to access/download;Figure;Figure_6.tif

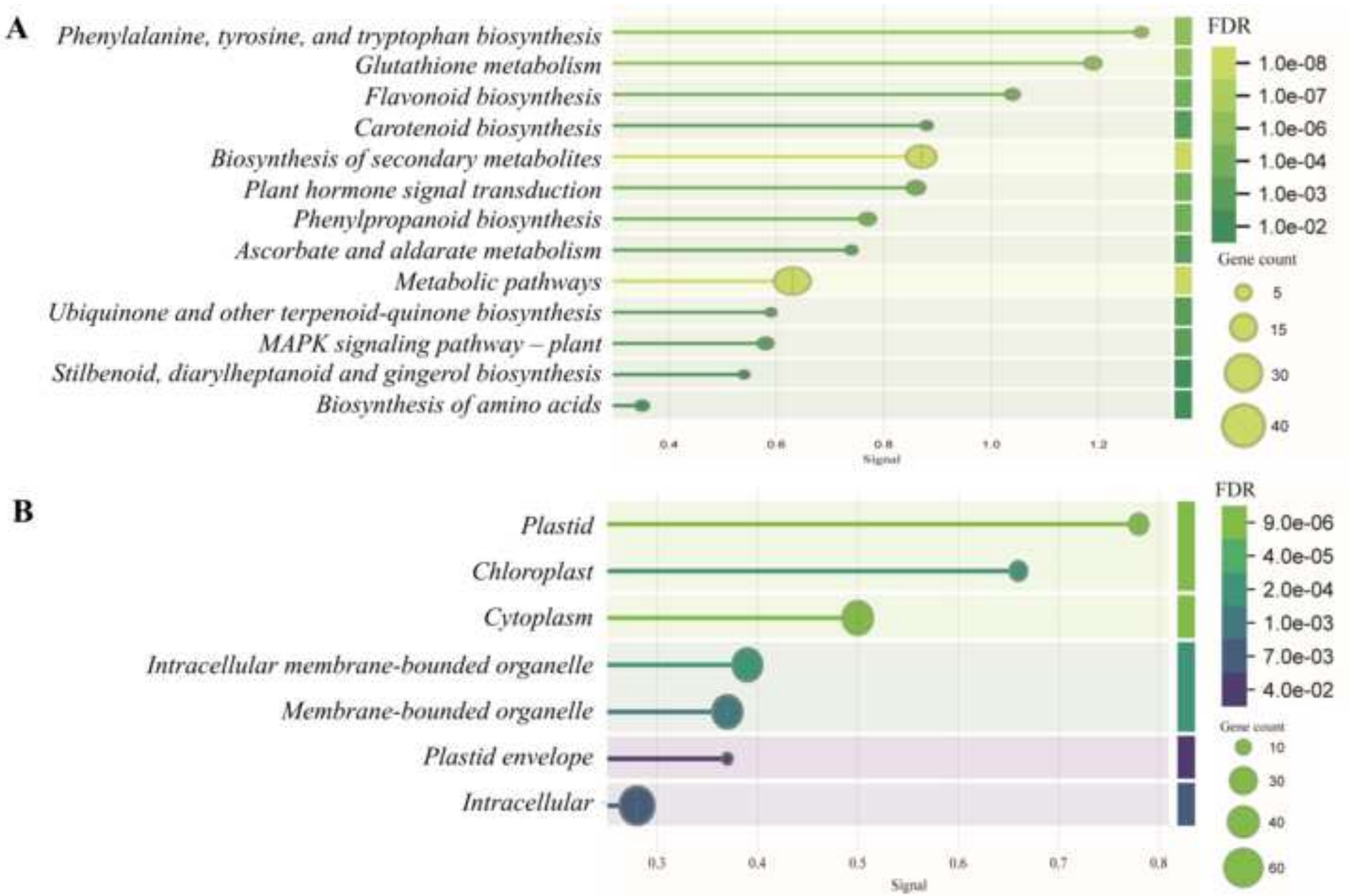

Click here to access/download

Supporting Information

Supplementary_File_S1.xlsx
